# Association Between Longer Cecal Intubation Time and Detection and Miss Rate of Colorectal Neoplasms

**DOI:** 10.3390/jcm13237080

**Published:** 2024-11-23

**Authors:** Ji Min Choi, Seon Hee Lim, Yoo Min Han, Jooyoung Lee, Eun Hyo Jin, Ji Yeon Seo, Jung Kim

**Affiliations:** Department of Internal Medicine, Healthcare Research Institute, Seoul National University Hospital Healthcare System Gangnam Center, Seoul 06236, Republic of Korea; 65846@snuh.org (J.M.C.); uminy@snuh.org (Y.M.H.); cheeryoung@hanmail.net (J.L.); icetea@snuh.org (E.H.J.); sjy@snuh.org (J.Y.S.); 83411@snuh.org (J.K.)

**Keywords:** adenoma detection rate, colonoscopy, cecal intubation time, colorectal neoplasms

## Abstract

**Background/Aims:** A longer cecal intubation time (CIT) occurs during colonoscopy under difficult insertion conditions, which may hinder meticulous mucosal observation. However, whether a longer CIT has detrimental effects on the detection of adenomas remains unclear. We evaluated the effects of CIT on the detection and miss rates of colorectal neoplasms in asymptomatic participants. **Methods:** Healthy examinees who underwent colonoscopy between March and July 2011, August 2015, and December 2016 were retrospectively enrolled. The primary outcome was the adenoma detection rate (ADR) across CIT quartiles, while the secondary outcomes included the mean number of adenomas, advanced ADR (AADR), clinically significant serrated lesion (CSSP) detection, adenoma miss rate (AMR), miss rate of CSSPs and any colorectal neoplasms, and the mean number of missed colorectal neoplasms in relation to CIT. **Results:** Overall, 12,402 participants were classified into quartiles according to the CIT. The longer the CIT, the lower the ADR (*p* < 0.001), AADR (*p* = 0.004), and mean number of adenomas (*p* < 0.001). The CSSP detection rate was not associated with CIT. On follow-up colonoscopy, AMR showed marginal increase with longer CIT (*p* = 0.065). The missed rates of CSSPs (*p* = 0.002) and colorectal neoplasms (*p* = 0.001) also increased with longer CIT. In the multivariate analysis, CIT was significantly associated with ADR, AADR, and AMR. **Conclusions:** Longer CIT was associated with lower ADR and higher AMR. Meticulous inspection is important for high-quality colonoscopy, particularly in patients requiring a longer CIT.

## 1. Introduction

Colorectal cancer (CRC) is the third most common malignancy and ranks fourth in cancer-related mortality worldwide [1]. Approximately 80% of CRCs originate from precursor adenomatous polyps; this carcinogenic process termed the adenoma–carcinoma sequence, typically takes years to decades [2]. Therefore, early detection and removal of premalignant polyps can significantly decrease the incidence of and mortality owing to CRC [3]. Although colonoscopy is the gold standard for CRC screening, recent studies have reported that the adenoma miss rate (AMR) of colonoscopy reaches 26%, which may be an important cause of post-colonoscopy colorectal cancer (PCCRC) [1]. Therefore, the adenoma detection rate (ADR) should be increased for effective colonoscopy.

The recommended target for ADR in asymptomatic average-risk individuals is ≥25% (≥30% for men and ≥20% for women), and each 1.0% increase in ADR reduces the incidence of CRC by 3.0% [4]. Current evidence suggests that various factors influence ADRs. These include patient-specific factors, such as age, sex, and comorbidities, and procedure-related factors, such as quality of bowel preparation, sufficient colonoscopy withdrawal time (CWT; >6 min), dynamic position changes in patients, additional examination of the right colon, and routine rectal retroflexion [5,6,7,8,9,10].

Cecal intubation time (CIT) is defined as the amount of time until the tip of the colonoscope reaches the cecum from the rectum [11]. Patients with the following factors (predictors of a longer CIT) tend to have more redundant and tortuous colons with marked loop formation: old age, female sex, low body mass index (BMI) or waist circumference, low visceral fat amount, constipation, and a history of abdominal surgery [11,12,13,14,15]. At the patient level, these anatomical limitations may make it difficult to straighten the colon, leading to insufficient exposure of the colonic mucosa and reduced lesion detection during colonoscopy [12]. At the endoscopist level, a challenging colonoscopy may interfere with securing sufficient CWT for polyp detection by increasing the physical fatigue and emotional burden of the endoscopist and causing time pressure. However, studies on the effects of CIT on ADRs are limited. Some studies have suggested that the shorter the CIT, the higher the ADR, whereas others have failed to establish a direct relationship between the two [10,16,17,18,19,20,21].

In this retrospective study, we investigated the association between the CIT and the detection or missed detection of colorectal neoplasms, including adenomas and serrated polyps, in asymptomatic healthy participants. We hypothesized that a longer CIT is associated with a lower detection rate and a higher miss rate of colorectal neoplasms. A clear understanding of this relationship is essential for high-quality colonoscopy.

## 2. Materials and Methods

### 2.1. Study Participants

**Figure 1:**
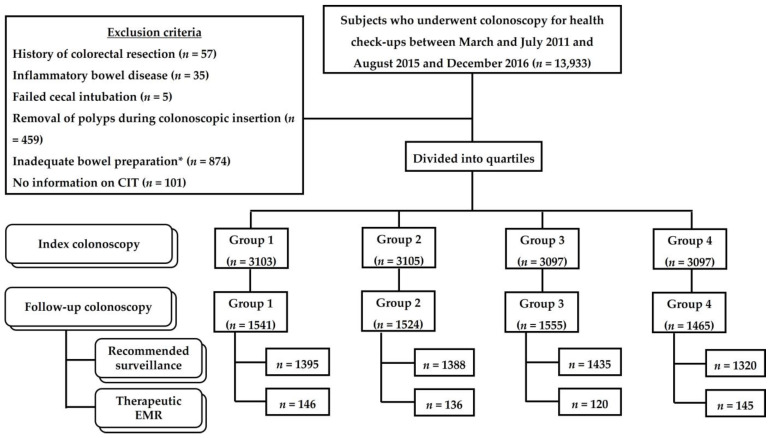
Flow diagram of the study population. * Aronchick scale ≥ 4 or segmental Boston bowel preparation scale ≤ 1; CIT, cecal intubation time; EMR, endoscopic mucosal resection.

Figure 1 illustrates the study design. Participants aged ≥ 18 years who underwent general health check-ups, including colonoscopy, at the Seoul National University Hospital (SNUH) Healthcare System Gangnam Center between March and July 2011 and August 2015 and December 2016 were screened for this retrospective study. Although our institution started officially recording the CIT and CWT on the endoscopy record sheet in August 2015, participants from March to July 2011 were also included in the analysis because, during this period, the procedure time was recorded on a trial basis [11]. The exclusion criteria were a prior history of colorectal resection or inflammatory bowel disease, failed cecal intubation, removal of polyps during colonoscope insertion, inadequate bowel preparation (Aronchick scale score of 4 or 5 and any segmental Boston bowel preparation scale [BBPS] score of 0 or 1), and no information on CIT. This study was reviewed and approved by the Ethics Committee of SNUH (IRB No. H-1903-045-1017) and was conducted in accordance with the Declaration of Helsinki. The requirement for written informed consent was waived owing to the retrospective nature of the study.

### 2.2. Study Design and Colonoscopy Procedures

Before 2015, bowel preparation was performed by oral administration of 4 L of polyethylene glycol solution (Colonlyte; Meditech Korea Pharmacy, Seoul, Republic of Korea). In March 2015, our institution introduced a split-dose regimen for bowel preparation, in which half of the bowel cleansing product (1 L of polyethylene glycol/ascorbic acid solution plus 0.5 L water; Coolprep^®^, Taejoon Pharm, Seoul, Republic of Korea) is administered the day before colonoscopy and the remainder is administered early in the morning on the day of colonoscopy. Further details of the split-dose regimen have been previously reported [22]. To assess bowel cleanliness, the Aronchick bowel preparation scale was used for participants examined in 2011 [11], and the BBPS was used for participants examined from 2015 onward [22].

The colonoscopy procedures were performed by 16 board-certified endoscopists who had performed >2000 colonoscopies, with cecal intubation rates of >98%. After cecal intubation, removable polyps were removed using biopsy forceps during withdrawal of the colonoscope, whereas participants with large polyps (≥1 cm) or cancer were referred to SNUH after biopsy of the lesion for second-stage endoscopic removal, such as endoscopic mucosal resection (EMR) or dissection, at the earliest. The endoscopists recorded the bowel preparation scale scores, CIT, CWT, and the need for position change and/or abdominal compression during insertion and provided a description of the polyp on the examination result sheet. CIT was defined as the time elapsed from insertion into the rectum to cecal intubation. CWT was defined as the time from cecal identification to the time when the colonoscope was withdrawn across the anus and included the time taken for biopsy. Endoscopists took and saved images of the landmarks during each colonoscopy. After completing the examination, the endoscopist calculated the time gap recorded in the image and recorded the CIT and CWT on the endoscopy sheet. Advanced adenoma was defined as tubular adenoma ≥ 1 cm, any adenoma containing villous histological features, or an adenoma with high-grade dysplasia. A clinically significant serrated polyp (CSSP) was defined as a sessile-serrated adenoma/polyp, a traditional serrated adenoma, or a hyperplastic polyp > 1 cm anywhere in the colon or >5 mm proximal to the sigmoid colon [23,24]. The polyp size was estimated by comparing it with closed or open biopsy forceps and the size of the pathologic report after the EMR. Our institution’s recommendations for follow-up colonoscopy were based on the American Gastroenterological Association guidelines [25]. PCCRC was defined as CRC diagnosed after a colonoscopy in which no cancer was detected [26].

### 2.3. Outcomes

The primary outcome was the ADR, which was defined as the percentage of participants with ≥1 adenoma during colonoscopy. One of the secondary outcomes was the AMR, which was defined as the percentage of participants with ≥1 missed adenoma during follow-up colonoscopy. The most reliable method for evaluating the AMR is “tandem” or “back-to-back” colonoscopy, in which two same-day colonoscopies are performed for each patient. However, in reality, these methods are difficult; thus, we defined the following as missed adenomas: (i) an adenoma > 5 mm found on the follow-up colonoscopy performed within the recommended surveillance interval, or (ii) any adenoma(s) found on repeat colonoscopy performed within 12 months due to the necessity of second-stage EMR, but not during baseline colonoscopy. We defined a missed CSSP as follows: (i) a sessile-serrated adenoma/polyp, traditional serrated adenoma > 5 mm, or hyperplastic polyp > 1 cm anywhere in the colon or >5 mm proximal to the sigmoid colon on follow-up colonoscopy performed within the recommended surveillance interval; or (ii) any CSSP found on repeat colonoscopy performed within 12 months due to the necessity of second-stage EMR. As a secondary outcome, the CSSP miss rate was calculated according to the AMR. Adenoma per colonoscopy (APC; defined as the total number of adenomas divided by the total number of colonoscopies performed), ADR by segment, advanced ADR (AADR), and CSSP detection rates (CSSDR) according to the CIT were also included as secondary outcomes. For the AMR, participants with at least one follow-up colonoscopy, specified by a predetermined surveillance schedule or additional endoscopic procedures immediately scheduled for polyp removal, were included in the analysis. Participants who underwent surveillance colonoscopy beyond the recommended period were excluded from the AMR analysis.

### 2.4. Statistical Analysis

We categorized the CIT of all participants into quartiles (Q1–Q4) to determine the relationship between the CIT and outcomes. To analyze the outcome of each colon segment, the entire colon was divided into the right colon (cecum and ascending colon), transverse colon (hepatic flexure, transverse colon, and splenic flexure), and left colon (descending and sigmoid colon, and rectum). Categorical variables are expressed as numbers (percentages) and were compared using the chi-square test or Fisher’s exact test. Trends in ordinal data were evaluated by *p*-values for linear-by-linear associations. Continuous variables are expressed as means with standard deviation and were compared using analysis of variance or the Kruskal–Wallis test. The Jonckheere–Terpstra test was used to assess trends in the number of detected and missed adenomas and the total number of missed colorectal neoplasms across the CIT quartiles. Univariate analysis was performed to evaluate the associations between the CIT and colonoscopic outcomes, as well as other relevant factors. A multivariate logistic regression model was used, which included age, sex, and BMI, which are well-known factors that influence CIT. Odds ratios (ORs) and 95% confidence intervals (CIs) were used to estimate the association between the CIT quartiles and the ADR, AADR, and AMR. Statistical analyses were performed using IBM SPSS 22.0 (IBM SPSS Statistics for Windows, Version 22.0, IBM Corp., Armonk, NY, USA). A *p*-value < 0.05 was considered statistically significant.

## 3. Results

### 3.1. Baseline Characteristics

A total of 13,933 participants handled by 16 expert endoscopists were reviewed for inclusion in this study. Among them, 12,402 eligible participants were included in the analysis and divided into four groups according to the CIT. Table 1 summarizes the baseline characteristics of the participants and the colonoscopic procedures performed in each CIT group. The mean insertion times in groups 1, 2, 3, and 4 were 125.29, 191.18, 265.48, and 502.96 s, respectively. Among the patient-related factors, female sex, older age, lower BMI, lower waist circumference, and history of prior abdominal surgery were associated with a longer CIT. The longer the CIT, the higher the position change rate or abdominal compression during colonoscope insertion.

### 3.2. Adenoma and CSSP Detection According to the CIT at the Index Colonoscopy

Table 2 presents the numbers and detection rates of various types of colorectal neoplasms, including pathologically proven adenomas, advanced adenomas, and CSSPs, stratified according to the CIT group. The ADR in this study was 36.7% (4548/12,402), which was significantly different according to the CIT group. When the entire colon was evaluated, the longer the CIT, the lower the ADR (40.9%, 36.3%, 35.5%, and 34.0% for groups 1, 2, 3, and 4, respectively; *p* < 0.001), even when dividing the colon into right, transverse, and left colon segments. The absolute number of detected adenomas per colonoscopy was significantly smaller in the longer CIT group than in the shorter CIT group (0.71, 0.61, 0.58, and 0.57 for groups 1, 2, 3, and 4, respectively; *p* < 0.001). In the shorter CIT group, the AADR was significantly higher than that in the longer CIT group (*p* = 0.004), while no significant difference was observed in the CSSDR according to the CIT (*p* = 0.657).

### 3.3. Adenoma and Serrated Lesion Detection Using the CIT at Follow-Up Colonoscopy

Among the 12,402 included participants, those who underwent follow-up colonoscopy (colonoscopy for second-stage EMR or performed within the recommended surveillance interval) at our institution were investigated. Consequently, 6085 patients (49.1%) were included in the follow-up colonoscopy analysis (Table 3). The missed ADR at the index colonoscopy (>5-mm adenoma found on follow-up colonoscopy, or any adenoma found in EMR cases) was 8.3% (508/6085) and marginally increased with the CIT (*p* = 0.065). This difference was particularly significant in the right colon, with no significant difference in the size distribution of the adenomas. In the longer CIT group, the missed CSSP detection rate was significantly higher (*p* = 0.002), particularly in the right colon and for CSSPs > 5 mm. Additionally, to understand the overall miss rate of clinically significant colon neoplasms, the detection rates of any missed colorectal neoplasms, including missed adenomas and CSSPs, in each group were compared. The longer the CIT, the significantly higher the detection rate of any missed colorectal neoplasms (*p* = 0.001). When comparing the absolute number of lesions found on follow-up colonoscopy, the number of missed adenomas (*p* = 0.046) and CSSPs (*p* = 0.002) increased significantly in the longer CIT group. When analyzing participants who underwent the recommended surveillance, a total of four PCCRC cases were found (0, 1, 2, and 1 in groups 1, 2, 3, and 4, respectively), with no significant difference among the groups (*p* = 0.708; Table 3). The proportion of participants requiring additional EMR was significantly higher in the longer CIT group than in the shorter CIT group (*p* < 0.001).

### 3.4. Factors Affecting Adenoma Detection and Missing Rates

The effects of the CIT as well as patient-related factors on the ADR, AADR, and AMR were analyzed (Table 4). When age, sex, BMI, and prior abdominal surgery were adjusted, the CIT was significantly associated with the ADR, AADR, and AMR. The ADR was significantly reduced in group 2 (OR: 0.865; 95% CI: 0.776–0.964; *p* = 0.009), group 3 (OR: 0.844; 95% CI: 0.755–0.943; *p* = 0.003), and group 4 (OR: 0.815; 95% CI: 0.727–0.914; *p* < 0.001) compared to group 1. Older age, male sex, and higher BMI were also independently associated positive factors influencing ADR. The AADR was significantly reduced in group 2 (OR: 0.667; 95% CI: 0.479–0.927; *p* = 0.016), group 3 (OR: 0.616; 95% CI: 0.438–0.867; *p* = 0.005), and group 4 (OR: 0.635; 95% CI: 0.449–0.898; *p* = 0.010) compared to group 1. Regarding the AMR, the adjusted OR was significantly higher in group 3 (OR: 1.320; 95% CI: 1.009–1.726; *p* = 0.043) and group 4 (OR: 1.405; 95% CI: 1.066–1.851; *p* = 0.016) compared to group 1. Age, male sex, and higher BMI also showed a positive association with a higher AMR, whereas a history of previous abdominal surgery did not show a significant independent association with AMR.

## 4. Discussion

Colonoscopy is regarded as the gold standard for colorectal cancer screening. Satisfaction of colonoscopy quality indicators is essential for effective detection of CRC at an early stage. However, in clinical practice, it is known that colonoscopy does not always detect all polyps. Missed adenomas, in particular, may serve as potential precursors for interval cancers, underscoring their significant clinical importance. Many studies have examined factors influencing the detection rate of colorectal neoplasms, such as cecal intubation rate, CWT, and bowel preparation quality. However, there has been limited research on the impact of CIT, which is influenced by various patient- and endoscopist-related factors, on successful colonoscopy outcomes.

To the best of our knowledge, this is the first study to evaluate the subsequent outcomes of ongoing colonoscopy screening and surveillance in a large healthy cohort according to the CIT at baseline colonoscopy. We investigated the association between the CIT at index colonoscopy and AMR during follow-up and the ADR at index colonoscopy in asymptomatic healthy participants. We found that the longer the CIT, the lower the ADR and AADR, whereas the AMR showed a tendency to increase. After adjusting for various related factors, CIT was significantly correlated with ADR, AADR, and AMR.

Cecal intubation is an essential prerequisite for effective colonoscopy, and a longer CIT reflects a technically more difficult examination [27]. The correlation between CIT and colonoscopy indicators, including ADR, has been investigated with inconsistent results. Renteln et al. showed that prolonged CIT is associated with a decrease in the number of adenomas, whereas the overall ADR did not differ significantly, and AMR was not investigated [17]. Moreover, Kim et al. found that delayed insertion time was an independent factor for missed polyps (OR: 4.10, 95% CI: 2.14–7.86) but not for missed adenomas [28]. Recently, Hamada et al. reported an inverse correlation between CIT and the total number of detected adenomas rather than ADR [21]. In their study, no significant correlation was observed between the detection of adenomas by size and advanced pathology based on CIT. Furthermore, this study included only female patients. However, some studies have shown that CIT is not related to ADR, polyp detection rate, or AMR [19,20,29]. Indeed, Fritz et al. found no correlation between the CIT and the mean number of adenomas or advanced adenomas per patient, but did demonstrate that the CIT-to-CWT ratio, rather than the CIT itself, was related to the ADR [19]. Another previous study also showed that the effect of CIT on the detection of adenomas was restricted to smaller adenomas that were relatively difficult to visualize [16].

BMI showed an inverse correlation with CIT in Table 1 of this study, suggesting an expected inverse correlation between BMI and AMR as well; however, multivariable analysis with various confounding factors, including CIT, revealed a positive correlation between them. In previous studies on predictive factors for missed adenoma, BMI has been reported as an independent predictor for any missed adenoma on repeat examination [30]. Thus, we speculate that the positive effect of BMI itself on AMR is stronger than the AMR reduction effect of CIT shortening, leading to the results observed in this study.

In this study, we found that a shorter CIT was associated with ADR, APC, and AADR at index colonoscopy but not with CSSP detection (*p* = 0.657). On follow-up colonoscopy, the CIT was significantly associated with the CSSP miss rate (*p* = 0.002) and showed a marginal association with AMR. Furthermore, we found a significant association when analyzing the association of CIT with all missed colorectal neoplasms, including adenomas and CSSPs. A longer CIT was also associated with a higher proportion of patients who required an additional procedure to remove large polyps after the recommended follow-up colonoscopy. This finding suggests that the longer the CIT, the greater the number of lesions with precancerous potential that are not detected during colonoscopy, representing an important clinical issue. The effect of CIT on adenoma detection and miss rates was significant even after adjusting for age, sex, and BMI. However, these findings should be interpreted with caution, given that CSSPs have a low detection rate at both index and follow-up colonoscopy. Hsieh et al. demonstrated that the issue of reduced ADR due to a longer CIT could be overcome by a relatively longer CWT [31].

In this study, we did not analyze the CWT by group because we could not collect the data about exact ‘CWT’. At our hospital, we recorded the time from reaching the cecum to the end of withdrawal, including the time elapsed during tissue biopsy or polyp removal. The pure “observation time”, not the total withdrawal time, is considered an important indicator for meticulous observation of the mucosa and detection of polyps. To compensate for the long observation times due to polyp removal, the median time elapsed during polyp removal was subtracted from the overall withdrawal time according to the number of polyps in individual examinees, as previously outlined [22]. As a result, the estimated observation time for each group did not differ significantly between groups (464.14 ± 163.60, 464.38 ± 168.51, 465.85 ± 185.22, and 481.20 ± 204.27 s for groups 1, 2, 3, and 4, respectively; *p*=0.279). Therefore, the difference in the CWT is unlikely to impact the difference in the adenoma detection and miss rate according to the CIT group.

Based on the findings of this study, it would be beneficial to discuss practical strategies that could help maintain ADR and reduce AMR in cases with longer CIT. First, it is essential to achieve sufficient exposure of the colonic mucosa by overcoming the anatomical limitations of the colon that require longer CIT. For cases where prolonged CIT is anticipated based on prior procedures, cap-assisted colonoscopy could facilitate with both insertion and withdrawal. Allocating a longer observation time than the generally required 6 min or keeping segmental withdrawal time longer than 2 min may be beneficial. In a recent Korean study, the ADR was significantly higher when the segmental withdrawal time was ≥2 min in the right-sided colon, ≥4 min in the proximal colon, and ≥3 min in the left-sided colon than when the segmental withdrawal times were shorter [32]. Additionally, when available, advanced imaging technologies such as narrow-band imaging or artificial intelligence (AI)-based computer-assisted detection systems (CADs) can be used to improve visualization and detection accuracy to maintain optimal colonoscopy results. AI-based CADs are promising, but can face obstacles such as accessibility, cost, and the need for additional training. Currently, these systems are only realistically applicable to a limited number of hospitals. Next, mitigating observer fatigue is also a challenging problem. In cases of longer CIT, consider having a second trained observer or tailored endoscopy technicians to help actively scanning the monitor for potential lesions. However, employing additional staff may not always be feasible due to financial constraints. Implementing short, scheduled breaks after challenging procedures can help reduce fatigue and maintain alertness, though this could impact patient scheduling and workflow. Further large multi-center prospective randomized controlled studies would be needed to investigate the role of structured rest, second trained observers, or advanced imaging techniques when cecal intubation is particularly difficult, and to provide a balanced view of the proposed strategies, considering both their benefits and potential limitations.

Our study had several strengths. First, we included a large number of participants and analyzed consecutive unselected participants who underwent routine colonoscopy screening, which increased the robustness of the results. Second, the endoscopists collected detailed covariate information regarding patient- and procedure-related factors, which allowed adjustment for other influencing factors. Third, the colonoscopy was performed by board-certified, highly experienced endoscopists (who performed more than 2000 colonoscopies) who satisfied the high-quality endoscopy criteria, including a cecal intubation rate > 98%, mean CWT > 6 min, and ADRs higher than the documented guideline of 25%. Additionally, their mean CIT was 271.10 ± 185.22 s, which is shorter than those outlined in previous studies [5,27,33,34,35]. Fourth, unlike other studies that primarily focused on adenomas, we included the detection and miss rates of serrated polyps, which are important colorectal neoplasms.

This study has several limitations. First, we recorded the CWT as the time from reaching the cecum to the end of withdrawal, including the time elapsed during tissue biopsy or polyp removal. The retrospective study design made it impossible to measure the inspection time alone. The precise effects of CWT should be verified in a prospective trial. Second, approximately half of the participants were lost to follow-up during the subsequent surveillance examinations. Because we analyzed participants who underwent voluntary health check-ups at our institution, follow-up was not compulsory. Third, the reasons for follow-up colonoscopy varied between participants (surveillance or treatment). Endoscopists may neglect to detect small polyps other than the target polyps when performing therapeutic endoscopy. Fourth, not all large polyps found during follow-up colonoscopy could be considered previously missed polyps. However, we considered this consistent because the lesions were classified by applying the same criteria to “missed adenomas or polyps” in each group. Fifth, assuming that the 16 endoscopists were at the same level, we grouped all endoscopists together without considering their individual results. The participating endoscopists are highly qualified, and our institution regularly conducts performance monitoring, including ADR and cecal intubation rates, to control endoscopist-specific variability. Nonetheless, the individual median CIT and ADR showed variation among endoscopists (Supplementary Figure S1). Given the technical differences among individuals, the observation of differences in absolute values among endoscopists was expected; however, further analysis using individual median CIT showed a trend toward lower ADRs and higher AMRs with longer CITs compared to shorter CITs (Supplementary Figures S2 and S3), consistent with the overall results of this study. Finally, this study was conducted at a single center with a high standard of endoscopy performance, so generalizing these results may require caution. Outcomes may differ between institutions, particularly where endoscopists have a broader range of experience levels. Institutions with less-experienced endoscopists or varying adherence to standard protocols might observe different results, potentially due to differences in procedural skills. Further large prospective multi-center studies, including the analysis based on experience level are needed in diverse clinical settings with endoscopists of varying skill and experience levels to verify the generalizability of our findings.

## 5. Conclusions

In conclusion, CIT was inversely correlated with ADR, AADR, and APC but had a proportional relationship with AMR. CIT was an independent factor that significantly affected ADR and AMR. Therefore, meticulous inspection for adenoma detection is important in colonoscopies with longer CITs. Endoscopists should ensure that they do not overlook adenomas by increasing the withdrawal time or by overcoming observer fatigue with various potential strategies, when performing colonoscopies for patients who require a long CIT. Further studies are required to establish whether missed adenomas can be prevented with sufficient CWT in long CIT groups.

## Figures and Tables

**Table 1:** Baseline Characteristics of the Study Participants and Colonoscopic Procedures.

	Quartiles of CIT				
	Group 1 (*n* = 3103)	Group 2 (*n* = 3105)	Group 3 (*n* = 3097)	Group 4 (*n* = 3097)	*p*-Value
Sex, male	2437 (78.5)	1899 (61.2)	1583 (51.1)	1267 (40.9)	<0.001
Age (years)	49.85 ± 9.92	50.94 ± 10.41	51.99 ± 10.60	52.54 ± 10.93	<0.001
Body mass index (kg/m^2^)	24.52 ± 3.08	23.60 ± 3.15	23.04 ± 3.15	22.55 ± 3.16	<0.001
Waist circumference (cm)	87.60 ± 8.47	84.76 ± 9.03	83.06 ± 9.15	81.72 ± 9.38	<0.001
Prior abdominal surgery	559 (18.0)	775 (25.0)	856 (27.6)	1011 (32.6)	<0.001
Mean insertion time (s)	125.29 ± 24.00	191.18 ± 18.38	265.48 ± 26.41	502.96 ± 233.00	<0.001
Position change during colonoscopy	222 (7.2)	402 (13.2)	706 (23.6)	1789 (59.9)	<0.001
Abdominal compression during colonoscopy	412 (13.7)	700 (24.1)	986 (36.1)	1659 (63.9)	<0.001

Values are presented as mean ± SD or n (%). CIT, cecal intubation time.

**Table 2:** Colorectal Neoplasm Detection According to the CIT at the Index Colonoscopy.

	Quartiles of CIT				
	Group 1 (*n* = 3103)	Group 2 (*n* = 3105)	Group 3 (*n* = 3097)	Group 4 (*n* = 3097)	*p*-Value
ADR					
Total colon	1269 (40.9)	1128 (36.3)	1098 (35.5)	1053 (34.0)	<0.001
Right colon	482 (15.5)	421 (13.6)	369 (11.9)	342 (11.0)	<0.001
Transverse colon	599 (19.3)	482 (15.5)	411 (13.3)	381 (12.3)	<0.001
Left colon	607 (19.6)	472 (15.2)	439 (14.2)	397 (12.8)	<0.001
APC	0.71 ± 1.16	0.61 ± 1.07	0.58 ± 1.05	0.57 ± 1.02	<0.001
AADR	93 (3.0)	62 (2.0)	58 (1.9)	59 (1.9)	0.004
CSSDR	115 (3.7)	115 (3.7)	111 (3.6)	123 (4.0)	0.657

Values are presented as the mean ± SD or n (%). CIT, cecal intubation time; ADR, adenoma detection rate; APC, adenoma per colonoscopy; AADR, advanced adenoma detection rate; CSSDR, clinically significant serrated polyp detection rate.

**Table 3:** Colorectal Neoplasm Detection According to the CIT at Follow-up Colonoscopy.

	Quartiles of CIT				
	Group 1 (*n* = 3103)	Group 2 (*n* = 3105)	Group 3 (*n* = 3097)	Group 4 (*n* = 3097)	*p*-Value
Participants who underwent follow-up colonoscopy	1541 (49.7)	1524 (49.1)	1555 (50.2)	1465 (47.3)	
Recommended surveillance	1395 (45.0)	1388 (44.7)	1435 (46.3)	1320 (42.6)	
Therapeutic EMR	146 (4.7)	136 (4.4)	120 (3.9)	145 (4.7)	
AMR	112 (7.3)	127 (8.3)	136 (8.7)	133 (9.1)	0.065
Location					
Right colon	36 (2.3)	40 (2.6)	57 (3.7)	53 (3.6)	0.013
Transverse colon	40 (2.6)	49 (3.2)	48 (3.1)	47 (3.2)	0.383
Left colon	48 (3.1)	54 (3.5)	46 (3.0)	58 (4.0)	0.361
Size					
Diminutive (≤5 mm)	19 (1.2)	27 (1.8)	24 (1.5)	23 (1.6)	0.573
Small (6–9 mm)	84 (5.5)	88 (5.8)	93 (6.0)	93 (6.3)	0.286
Large (≥10 mm)	19 (1.2)	20 (1.3)	25 (1.6)	26 (1.8)	0.168
CSSP miss rate	36 (2.3)	41 (2.7)	57 (3.7)	60 (4.1)	0.002
Location					
Right colon	17 (1.1)	24 (1.6)	34 (2.2)	32 (2.2)	0.010
Transverse colon	13 (0.8)	16 (1.0)	18 (1.2)	23 (1.6)	0.064
Left colon	8 (0.5)	5 (0.3)	8 (0.5)	8 (0.5)	0.742
Size					
Diminutive (≤5 mm)	5 (0.3)	3 (0.2)	2 (0.1)	2 (0.1)	0.212
Small (6–9 mm)	25 (1.6)	28 (1.8)	39 (2.5)	39 (2.7)	0.023
Large (≥10 mm)	8 (0.5)	15 (1.0)	17 (1.1)	22 (1.5)	0.008
Any colorectal neoplasms miss rate	140 (9.1)	162 (10.6)	181 (11.6)	187 (12.8)	0.001
Number of missed adenomas	0.09 ± 0.33	0.11 ± 0.47	0.11 ± 0.46	0.12 ± 0.44	0.046
Number of missed CSSPs	0.03 ± 0.20	0.03 ± 0.19	0.04 ± 0.24	0.05 ± 0.24	0.002
PCCRC detection rate *	0 (0)	1 (0.1)	2 (0.1)	1 (0.1)	0.708
Necessity of additional EMR *	41 (2.9)	55 (4.0)	73 (5.1)	74 (5.6)	<0.001

Values are presented as the mean ± SD or n (%). * Analyzed participants who underwent the recommended surveillance. CIT, cecal intubation time; EMR, endoscopic mucosal resection; AMR, adenoma miss rate; CSSP, clinically significant serrated polyp; PCCRC, post-colonoscopy colorectal cancer.

**Table 4:** Effect of the CIT and patient-related factors on the ADR, AADR and AMR.

	Adjusted OR (95% CI)	*p*-Value
ADR		
Age	1.061 (1.057–1.066)	<0.001
Sex, male	1.841 (1.681–2.016)	<0.001
BMI	1.046 (1.032–1.060)	<0.001
Prior abdominal surgery	1.023 (0.931–1.123)	0.636
CIT		
Group 1	1.000 (reference)	
Group 2	0.865 (0.776–0.964)	0.009
Group 3	0.844 (0.755–0.943)	0.003
Group 4	0.815 (0.727–0.914)	<0.001
AADR		
Age	1.052 (1.039–1.064)	<0.001
Sex, male	1.658 (1.233–2.230)	0.001
BMI	1.014 (0.972–1.059)	0.510
Prior abdominal surgery	1.016 (0.756–1.363)	0.918
CIT		
Group 1	1.000 (reference)	
Group 2	0.667 (0.479–0.927)	0.016
Group 3	0.616 (0.438–0.867)	0.005
Group 4	0.635 (0.449–0.898)	0.010
AMR		
Age	1.045 (1.035–1.055)	<0.001
Sex, male	1.989 (1.574–2.513)	<0.001
BMI	1.042 (1.008–1.077)	0.015
Prior abdominal surgery	1.152 (0.924–1.438)	0.209
CIT		
Group 1	1.000 (reference)	
Group 2	1.238 (0.945–1.620)	0.121
Group 3	1.320 (1.009–1.726)	0.043
Group 4	1.405 (1.066–1.851)	0.016

CIT, cecal intubation time; ADR, adenoma detection rate; AADR, advanced adenoma detection rate; AMR, adenoma miss rate; BMI, body mass index.

## Data Availability

The datasets generated during and/or analyzed during the current study are available from the corresponding author upon reasonable request.

## Supplementary Materials

The following supporting information can be downloaded at: https://www.mdpi.com/article/10.3390/jcm13237080/s1, Figure S1: Median CIT and ADR for individual endoscopists. CIT, cecal intubation time; ADR, adenoma detection rate; Figure S2: ADR according to individual endoscopists’ CIT. * *p*-value < 0.05. CIT, cecal intubation time; ADR, adenoma detection rate; Figure S3: AMR according to individual endoscopists’ CIT. All *p*-values > 0.05. CIT, cecal intubation time; AMR, adenoma miss rate.
