# Supplementary material for: Association Between Longer Cecal Intubation Time and Detection and Miss Rate of Colorectal Neoplasms
Source: J Clin Med. 2024 Nov 23;13(23):7080. doi: 10.3390/jcm13237080 (PMC11642371; doi:10.3390/jcm13237080)
Supplement: Supplementary file 1 [file jcm-13-07080-s001.zip › jcm-3278117-supplementary.pdf]

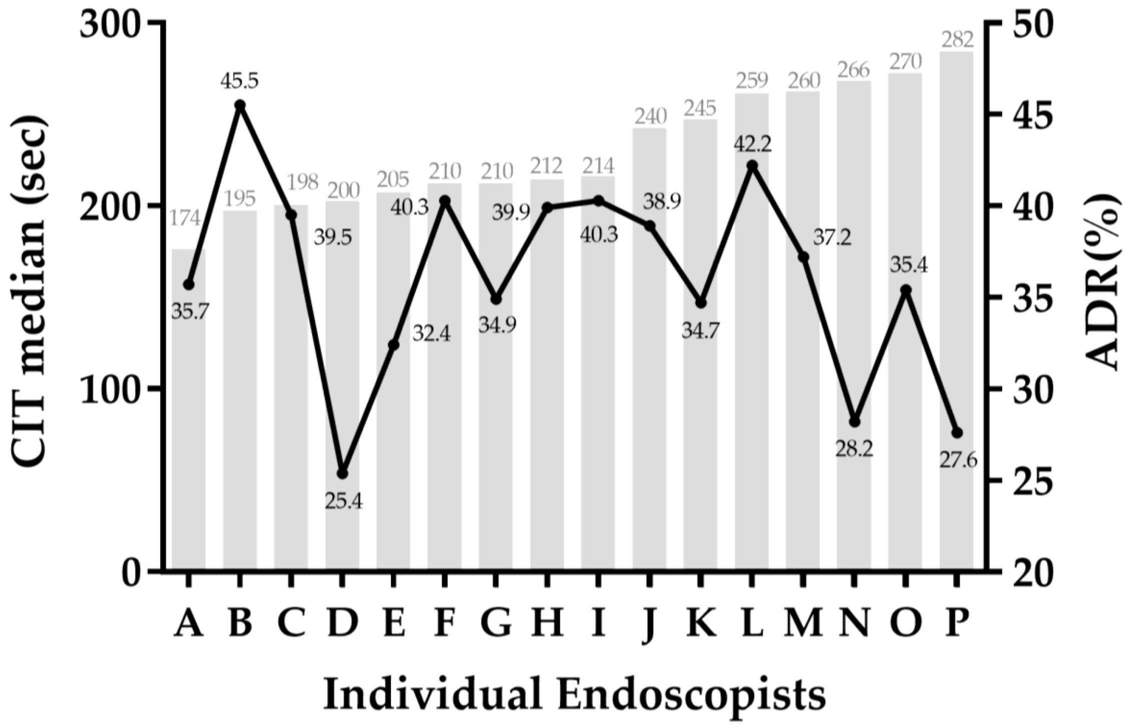

Supplementary Figure S1. Median CIT and ADR for individual endoscopists. CIT, cecal intubation time; ADR, adenoma detection rate.

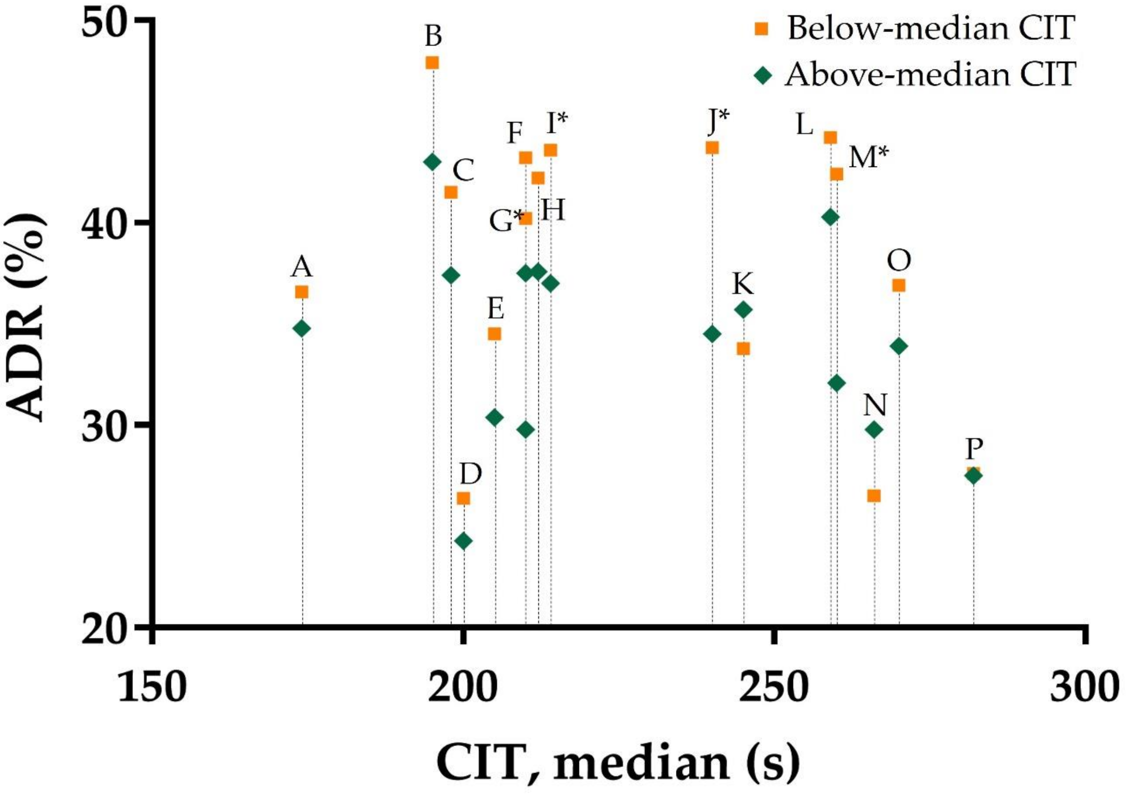

Supplementary Figure S2. ADR according to individual endoscopists' CIT. *P-value<0.05. CIT, cecal intubation time; ADR, adenoma detection rate.

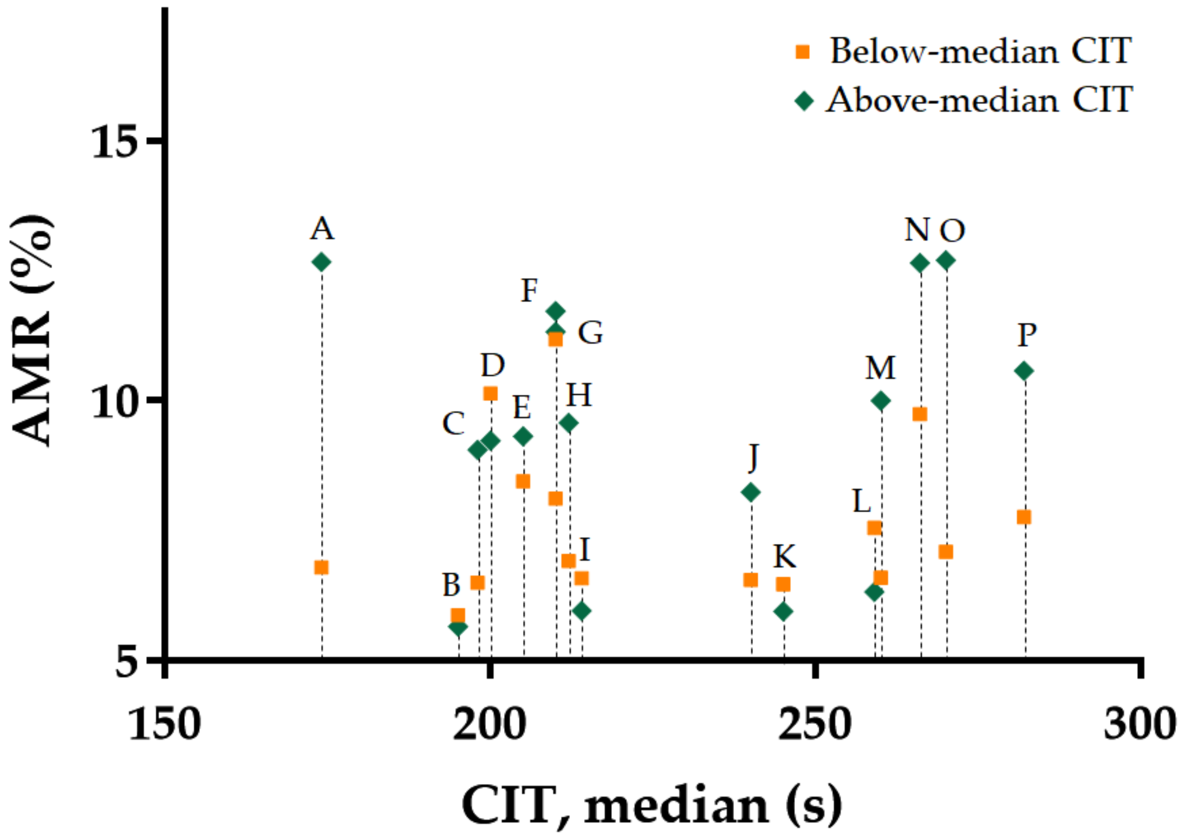

Supplementary Figure S3. AMR according to individual endoscopists' CIT. All *P*-values>0.05.

CIT, cecal intubation time; AMR, adenoma miss rate.
